# A study on the early metabolic effects of salt and fructose consumption: the protective role of water

**DOI:** 10.1038/s41440-024-01686-8

**Published:** 2024-05-15

**Authors:** Nuri Baris Hasbal, Cicek Nur Bakir, Said Incir, Dimitrie Siriopol, Laura G. Sanchez-Lozada, Miguel A. Lanaspa, Richard J. Johnson, Mehmet Kanbay

**Affiliations:** 1 Division of Nephrology, Department of Internal Medicine, Koc University School of Medicine, İstanbul, Turkey; 2 Koc University School of Medicine, Istanbul, Turkey; 3 Department of Biochemistry, Koc University School of Medicine, Istanbul, Turkey; 4 Department of Nephrology, “Saint John the New” County Hospital, Stefan cel Mare University, Suceava, Romania; 5 Department of Cardio-Renal Physiopathology, Instituto Nacional de Cardiología “Ignacio Chavez”, Mexico City, Mexico; 6 Department of Medicine, University of Colorado Anschutz Medical Center, Aurora, CO USA

**Keywords:** Salt, Fructose, Osmolality, Blood pressure

## Abstract

Increasing serum osmolality has recently been linked with acute stress responses, which over time can lead to increased risk for obesity, hypertension, and other chronic diseases. Salt and fructose are two major stimuli that can induce acute changes in serum osmolality. Here we investigate the early metabolic effects of sodium and fructose consumption and determine whether the effects of sodium or fructose loading can be mitigated by blocking the change in osmolality with hydration. Forty-four healthy subjects without disease and medication were recruited into four groups. After overnight fasting, subjects in Group 1 drank 500 mL of salty soup, while those in Group 2 drank 500 mL of soup without salt for 15 min. Subjects in Group 3 drank 500 mL of 100% apple juice in 5 min, while subjects in Group 4 drank 500 mL of 100% apple juice and 500 mL of water in 5 min. Blood pressure (BP), plasma sodium, and glucose levels were measured every 15 min in the first 2 h. Serum and urine osmolarity, serum uric acid, cortisol, fibroblast growth factor 21 (FGF21), aldosterone, adrenocorticotropic hormone (ACTH) level, and plasma renin activity (PRA) were measured at the baseline and 2 h. Both acute intake of salt or fructose increased serum osmolality (maximum ∼4 mOsm/L peaking at 75 min) associated with a rise in systolic and diastolic BP, PRA, aldosterone, ACTH, cortisol, plasma glucose, uric acid, and FGF21. Salt tended to cause greater activation of the renin-angiotensin-system (RAS), while fructose caused a greater rise in glucose and FGF21. In both cases, hydration could prevent the osmolality and largely block the acute stress response. Acute changes in serum osmolality can induce remarkable activation of the ACTH-cortisol, RAS, glucose metabolism, and uric acid axis that is responsive to hydration. In addition to classic dehydration, salt, and fructose-containing sugars can activate these responses. Staying well hydrated may provide benefits despite exposure to sugar and salt. More studies are needed to investigate whether hydration can block the chronic effects of sugar and salt on disease.

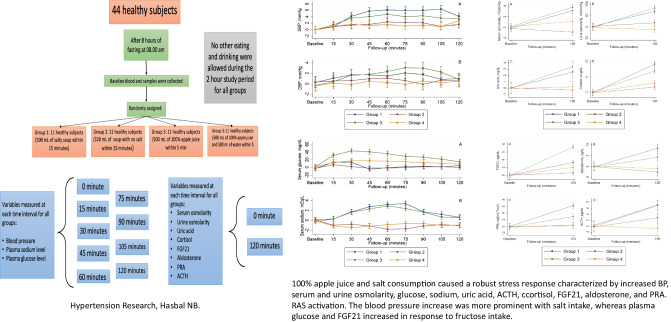

## Introduction

There is increasing evidence that even mild increases in serum osmolarity can be associated with increased risk for a variety of noncommunicable diseases, including hypertension [1], chronic kidney disease [2], heart failure [3], and even aging-associated diseases [4]. The mechanisms are complex but likely involve stimulation of the renin-angiotensin system (RAS), vasopressin, cortisol, and fructose-metabolism pathways [5–10].

While dehydration and heat stress are commonly known mechanisms for increasing serum osmolarity, salt, and sugar also have acute effects of increasing serum osmolarity, with the effects of sugar due to fructose. Indeed, there is evidence that the effects of salt to increase blood pressure (BP), at least acutely, is due to the change in serum osmolarity rather than the salt load [11, 12]. Besides, hydration that blocks the rise in serum sodium with an acute salt load, can block the rise in BP [12]. Hydration can also block salt-induced metabolic syndrome by blocking osmolarity-induced fructose generation through the polyol pathway [13].

Here, we performed a study to investigate further the effects of increasing serum osmolarity on acute stress responses and specifically to compare the effects of salt with fructose (the latter provided in 100% apple juice). This comparison is clinically important since sugar and salt are so commonly ingested in the Western diet. We found that both could induce a comparable rise in serum osmolarity with stress responses, though the salt caused a greater activation of the RAS, and fructose caused a greater response of the glucose and fibroblast growth factor 21 (FGF21) metabolism.

Point of view

- Clinical relevance
  this study highlights the critical need for hydration in mitigating the early metabolic effects of salt and fructose consumption, underscoring a straightforward yet effective strategy for preventing potential health risks associated with diet-induced changes in serum osmolarity.
- Future direction
  future research should explore long-term clinical trials focusing on the role of hydration in preventing the chronic effects of high salt and sugar diets, potentially leading to new dietary guidelines and interventions aimed at improving public health outcomes.
- Consideration of the Asian population
  given the dietary habits and climatic diversity across Asia, this study’s findings emphasize the importance of tailored public health strategies that account for regional dietary preferences and environmental factors to effectively manage the metabolic impact of salt and sugar consumption.

## Materials and methods

### Characteristics of the study population

Forty-four healthy individuals with body mass index (BMI) between 19–25 kg/m^2^ without any systemic diseases and using no medications were included in the study. All volunteers were asked not to eat or drink anything except water after 00.00 until the intervention at 08.00.

**Fig. 1:**
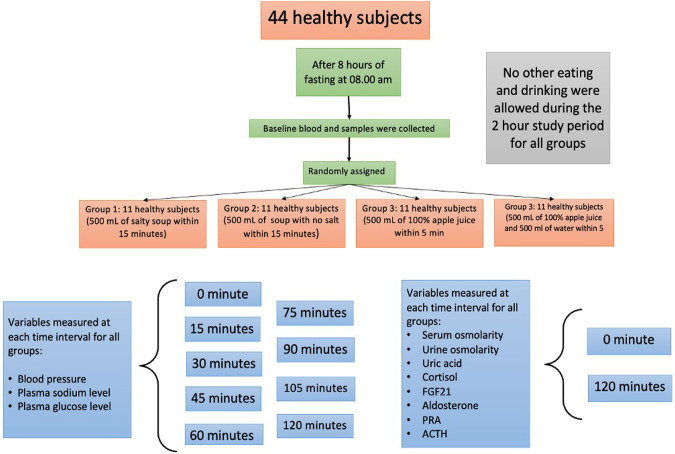
The study protocol

Baseline laboratory tests were performed before starting to drink soup or apple juice. Baseline measurements include urinalysis, serum adrenocorticotropic hormone (ACTH), serum cortisol, plasma renin activity (PRA), and serum and urine osmolarity. Starting at 0 min, BP was monitored every 15 min. Blood samples were also collected at these intervals to evaluate serum glucose and serum sodium. At 120 min, urine and blood samples were collected again to measure serum ACTH, serum cortisol, serum PRA, and serum and urine osmolarity. The study protocol is summarized in Fig. 1.

The Koc University School of Medicine ethics committee approved the study protocol (2022.009.IRB1.009). Written informed consent was taken from all participants before enrollment.

### Study protocol

Dietary salt intervention was studied by testing the effect of prescribed salt intake with 500 mL lentil soup for Group 1 and Group 2. The sodium and potassium contents of the lentil soup were 30 mg and 790 mg in 100 g lentils, respectively. A dietician prepared the 500 mL soup [for 100 mL, energy (Kcal): 45.6, fat (g): 0.20, saturates (g):0, carbohydrate (g): 8.28, sugar (g): 0, protein (g): 2.46, and fiber (g): 1.75] with an additional 3 g of salt (Group 1) and no additional salt (Group 2). Each participant in Group 1 and Group 2 drank the same 500 mL of soup within 15 min.

Dietary fructose intervention was studied by testing the effect of 500 mL of 100% pure, commercially available apple juice [for 100 mL, energy (Kcal): 48, fat (g): 0, saturates (g): 0, carbohydrate (g): 11.5, sugar (g): 10.3, protein (g): 0.5, fiber (g): 0, and salt: 0] for Group 3 and Group 4, respectively (https://www.isrctn.com/ISRCTN14798840). Hundred-percent apple juice was chosen as the source of fructose due to having a higher fructose-to-glucose ratio and having no antioxidant effect [14]. Each participant in Group 3 and Group 4 drank the same 500 mL of apple juice in 5 min, Group 4 additionally drank 500 mL of water.

No other eating or drinking was allowed during the 2-h study period. The subjects did not perform any physical activity during the study period.

### Serum measurements

Blood samples were collected into dry tubes and subsequently subjected to centrifugation at 3500 g for 10 min at +4 °C to isolate sera in aliquots. Cortisol and ACTH levels were promptly quantified in the separated sera utilizing the electrochemiluminescence immunoassay method. This analysis was conducted using a Roche Cobas Pro analyzer (Roche, Basel, Switzerland). Simultaneously, uric acid levels were determined through the application of the colorimetric method. The residual sera, not utilized during the immediate analysis, were meticulously preserved at −80 °C for subsequent examination. Serum osmolarity was evaluated using the freezing-point Osmometer K-7400S (Knauer, Berlin, Germany), which permits freezing-point depression to be assessed.

The competitive inhibition enzyme-linked immunosorbent assay technique was applied to measure FGF 21, aldosterone, and PRA concentrations (USCN, Wuhan, China). Intra- and inter-coefficients of variation for, FGF 21, aldosterone, and PRA tests were below 10% and 12%, respectively.

### BP measurements

Participants were included in the study after resting for 20 min. Throughout the study, they were not restricted in movement but were advised against performing strenuous physical activities, and due to intermittent BP measurement and blood sampling, they were asked not to leave the area where the study was conducted. BP was assessed using the Omron HEM 907 oscillometric monitor (Omron Healthcare), which was validated for use with an appropriately sized cuff for the participant’s arm. The BP measurements were conducted following a 5-min period of quiet rest while seated, with the arm positioned at heart level. Three consecutive measurements were taken, spaced 30 s apart, and the average of these three readings was recorded.

### Statistical analysis

Data are expressed as mean with standard deviation, number with percent frequency, or in the linear mixed models, as mean with 95% confidence interval (CI). For the demographic variables, between-group comparisons were assessed with the w2 test or one-way analysis of variance, as appropriate. The Shapiro–Wilk test was used to assess the distribution of the continuous variables. Time-repeated measurements were taken using linear mixed models including treatment, time, and the treatment by time interaction term. Since all the variables had a normal distribution, we analyzed them through mixed models for repeated measurements, adjusting for the baseline levels and for the demographic variables with values significantly different between the groups. Group inferences, effect estimates, and 95% CIs were taken from these models. At each time, the multiple group comparisons were corrected using Bonferroni’s method.

## Results

### Demographic information

As shown in Table 1, there were no differences regarding age and gender. However, individuals from Groups 3 and 4 had a higher BMI than individuals from Group 2; individuals from Group 4 also had a higher BMI than those from Group 1.

**Table 1:** Demographic and clinical characteristics of the different groups

	All, (*n* = 44)	Group 1, (*n* = 11)	Group 2, (*n* = 11)	Group 3, (*n* = 11)	Group 4, (*n* = 11)	*p* For trend
Age, years	30.5 ± 6.4	28.0 ± 5.7	31.2 ± 7.5	30.5 ± 5.5	32.2 ± 6.8	0.47
Male, *N* (%)	34 (77.3)	8 (72.7)	7 (63.6)	10 (90.9)	9 (81.8)	0.46
BMI, kg/m^2^	26.9 ± 2.1	25.4 ± 3.7	24.6 ± 3.7	28.6 ± 2.3	29.4 ± 2.1	0.001

Data are expressed as mean with standard deviation or number with percent frequency, as appropriate

Group 1: 500 mL salty soup, Group 2: 500 mL soup with no additional salt soup, Group 3: 500 mL fructose (apple juice), Group 4: 500 mL apple juice + 500 mL water

*BMI* body mass index

### The effect of interventions on BP levels

**Fig. 2:**
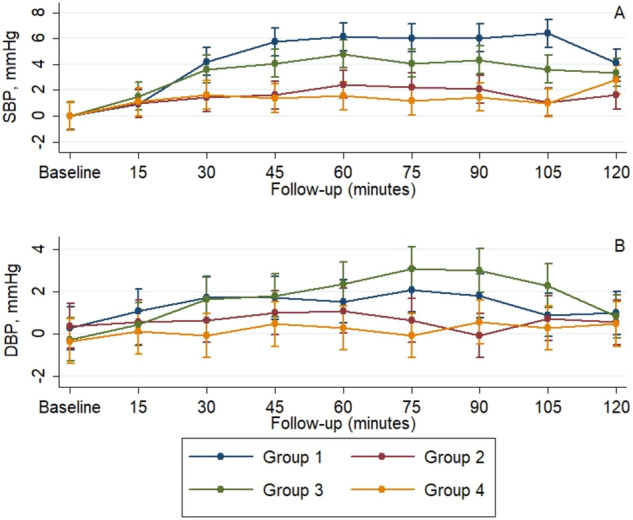
Blood pressures mean values across the follow-up. **A** Systolic blood pressure (SBP). **B** Diastolic blood pressure (DBP)

At baseline, there was no significant difference between the 4 groups in regard to systolic and diastolic BP values (Table 2). There was an increase in the systolic BP mean values across the follow-up (p* < 0.001), with a significant difference in the slope between the four groups (*p*^†^ < 0.001; see also Fig. 2A). At 30 min, as compared with the baseline values, there was a significant increase in systolic BP values only in individuals from Groups 1 and 3 (adjusted mean difference 4.2 mmHg, 95% CI 2.0–6.3 mmHg and 3.6 mmHg, 95% CI 1.5–5.8 mmHg, respectively), and remained significant the entire follow-up. No consistently significant differences were observed in individuals from Groups 2 and 4. After the initial 15 min, at each 15-min interval of follow-up interval, the mean values of systolic BP were higher in individuals from Group 1 compared to individuals from Groups 2 and 4. At 30 min of follow-up, the mean systolic BP levels in individuals from Group 3 were significantly higher than those observed in individuals from Group 2; after this, the systolic BP values were higher only as compared with those from individuals in Group 4.

**Table 2:** The effect of interventions on systolic and diastolic BP levels

	Length of follow-up									p*	*p*^†^
	Baseline	15 min	30 min	45 min	60 min	75 min	90 min	105 min	120 min		
Systolic BP, mmHg											
Group 1	113.7 (109.5–117.9)	114.6 (110.4–118.8)	117.9 (113.7–122.1)	119.5 (115.3–123.6)	119.8 (115.6–124.0)	119.7 (115.5–123.9)	119.7 (115.5–123.9)	120.1 (115.9–124.3)	117.8 (113.6–122.0)	<0.001	<0.001
Group 2	111.6 (107.4–115.8)	112.6 (108.4–116.8)	113.1 (108.9–117.3)	113.3 (109.1–117.5)	114.1 (109.9–118.3)	113.9 (109.7–118.1)	113.7 (109.5–117.9)	112.7 (108.5–116.9)	113.3 (109.1–117.5)		
Group 3	108.9 (104.7–113.1)	110.5 (106.3–114.6)	112.5 (108.4–116.7)	113.0 (108.8–117.2)	113.7 (109.5–117.9)	113.0 (108.8–117.2)	113.3 (109.1–117.5)	112.5 (108.4–116.7)	112.3 (108.1–116.5)		
Group 4	109.5 (105.3–113.6)	110.5 (106.4-114.7)	111.1 (106.9–115.3)	110.8 (106.6–115.0)	111.0 (106.8–115.2)	110.6 (106.4–114.8)	110.9 (106.7–115.1)	110.5 (106.3–114.6)	112.3 (108.1–116.5)		
*p*^‡^	0.36	0.88	<0.001	<0.001	<0.001	<0.001	<0.001	<0.001	0.01		
Diastolic BP, mmHg											
Group 1	71.5 (67.6–75.3)	72.3 (68.4–76.1)	72.9 (69.1–76.7)	72.9 (69.1–76.7)	72.7 (68.9–76.6)	73.3 (69.4–77.1)	73.0 (69.2–76.8)	72.1 (68.3–75.9)	72.2 (68.3–76.0)	<0.001	<0.001
Group 2	71.1 (67.3–74.9)	71.3 (67.4–75.1)	71.4 (67.5–75.2)	71.7 (67.9–75.6)	71.8 (67.9–75.7)	71.4 (67.5–75.2)	70.6 (66.8–74.5)	71.5 (67.6–75.3)	71.3 (67.4–75.1)		
Group 3	69.7 (65.9–73.6)	70.5 (66.6–74.3)	71.6 (67.8–75.5)	71.8 (67.9–75.7)	72.4 (68.5–76.2)	73.1 (69.3–76.9)	73.0 (69.2–76.8)	72.3 (68.4–76.1)	70.8 (66.9–74.7)		
Group 4	71.6 (67.8–75.5)	72.1 (68.3–75.9)	71.9 (68.1–75.7)	72.5 (68.6–76.3)	72.3 (68.4–76.1)	71.9 (68.1–75.7)	72.5 (68.7–76.4)	72.3 (68.4–76.1)	72.5 (68.6–76.3)		
* p*^‡^	0.89	0.65	0.04	0.21	0.04	<0.001	<0.001	0.04	0.88		

Data are presented as mean (95% CI) at baseline, and least-squares mean (95% CI) at baseline, 15 min, 30 min, 45 min, 60 min, 75 min, 90 min, 105 min, and 120 min, respectively

Analysis was conducted using a mixed model for repeated measures, adjusting for baseline values

*p Value for time effect—trend over time in all arms

^†^*p* Value for treatment × time interaction—evaluates if changes in one arm are different from the changes in the other arms

^‡^*p* Value for comparison between arms at each moment

Although there was an increase in the mean values across the follow-up, with a significant difference in the slope of the increase between the 4 groups (Table 2 and Fig. 2B), there were different patterns of evolution of diastolic BP mean values during the follow-up. Compared with baseline levels, only individuals from Group 3 showed a significantly consistent increase in the mean diastolic BP after the initial 30 min of follow-up. After 60 min of follow-up, the mean values of diastolic BP were higher in individuals from Group 3 as compared with individuals from Group 4. At the final assessment point, there was no difference between the four groups of individuals.

### The effect of interventions on serum and urine osmolarity

**Fig. 3:**
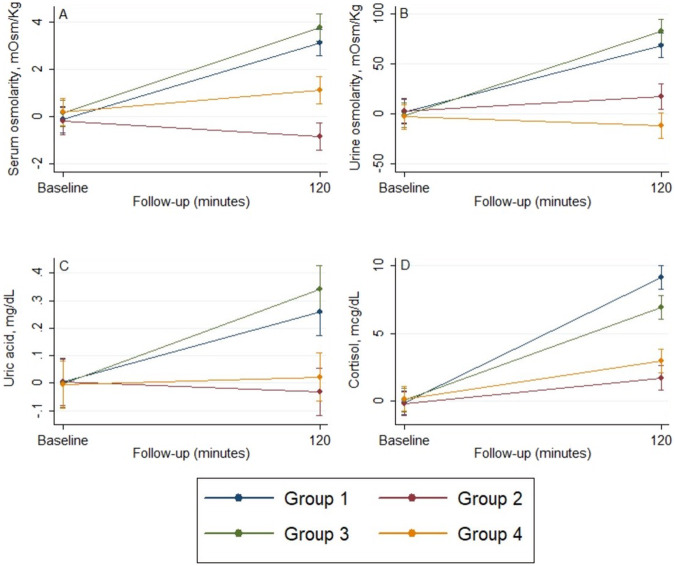
The mean values of study parameters across the follow-up. **A** Serum osmolarity. **B** Urine osmolarity. **C** Serum uric acid, **D** Serum cortisol

At baseline, there was no difference between the 4 groups of individuals regarding both serum and urine osmolarity (Table 3). There was a significant increase in the mean values in the 4 groups during the follow-up (p* < 0.001), and a significant difference in the slope of the increase between the 4 groups of individuals (*p*^†^ < 0.001; see also Fig. 3A, B). Individuals from Groups 1 and 3 significantly increased mean serum and urine osmolarity, and these values were significantly higher at 120 min than those observed in Groups 2 and 3.

**Table 3:** The effect of interventions on serum and urine osmolarity

	Length of follow-up			
	Baseline	120 min	p*	*p*^†^
Serum osmolarity, mOsm/Kg				
Group 1	289.0 (287.1–290.9)	292.3 (290.4–294.1)	<0.001	<0.001
Group 2	290.1 (288.2–291.9)	289.5 (287.6–291.3)		
Group 3	287.8 (285.9–289.7)	291.5 (289.6–293.3)		
Group 4	289.9 (288.0–291.8)	290.8 (288.9–292.7)		
* p*^‡^	0.31	<0.001		
Urine osmolarity, mOsm/Kg				
Group 1	314.0 (253.3–374.7)	380.5 (319.9–441.2)	<0.001	<0.001
Group 2	294.3 (233.6–354.9)	308.2 (247.5–368.8)		
Group 3	312.7 (252.1–373.4)	396.9 (336.3–457.6)		
Group 4	339.8 (279.2–400.5)	330.9 (270.3–391.6)		
* p*^‡^	0.78	<0.001		

Data are presented as mean (95% CI) at baseline and 2 h

Analysis was conducted using a mixed model for repeated measures, adjusting for baseline values

*p Value for time effect—trend over time in all arms

^†^*p* Value for treatment × time interaction—evaluates if changes in one arm are different from the changes in the other arms

^‡^*p* Value for comparison between arms at each moment

### The effect of interventions on serum sodium concentrations

**Fig. 4:**
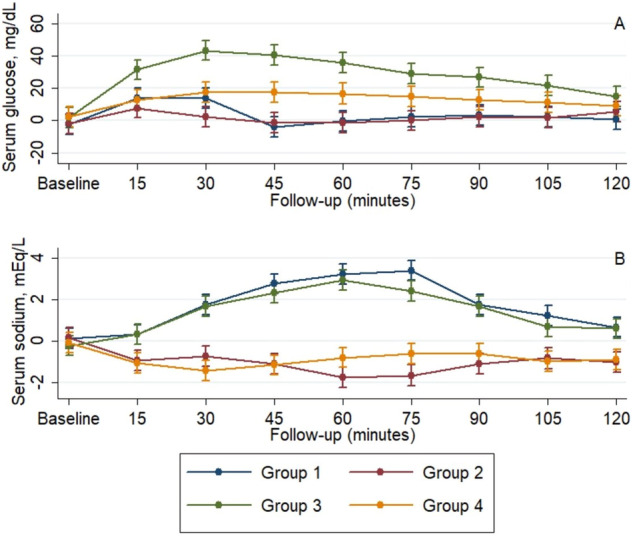
The mean values of serum parameters across the follow-up. **A** Glucose. **B** Sodium

The baseline serum sodium concentrations were similar between the 4 groups at baseline (Table 4). During the follow-up, considering all the groups, there was a significant increase in the mean plasma sodium concentration (Table 4, p* < 0.001) and a significant difference in the slope between the four groups (Table 4, *p*^†^ < 0.001). At 30 min, as compared with the baseline values, there was a significant increase in serum sodium values only in individuals from Groups 1 and 3 (adjusted mean difference 1.6 mEq/L, 95% CI 0.7–2.5 mEq/L mmHg and 1.9 mEq/L, 95% CI 1.0–2.8 mEq/L, respectively), that remained, except for the 120 min assessment, significant the entire follow-up (Fig. 4B). On contrast, as compared with baseline levels, individuals from Group 2 showed a significant decrease in mean serum sodium levels at all points of follow-up, while those in Group 4, only for the first 45 min of follow-up.

**Table 4:** The effect of interventions on serum sodium concentrations

	Length of follow-up									p*	*p*^†^
	Baseline	15 min	30 min	45 min	60 min	75 min	90 min	105 min	120 min		
Sodium, mmol/L											
Group 1	139.9 (139.0–140.8)	140.1 (139.2–140.9)	141.5 (140.7–142.4)	142.5 (141.7–143.4)	143.0 (142.1–143.9)	143.2 (142.3–144.1)	141.5 (138.6–144.5)	141.0 (140.7–142.4)	141.0 (140.1–141.9)	0.55	0.16
Group 2	140.1 (139.6–141.3)	139.0 (138.1–139.9)	139.2 (138.3–140.1)	138.8 (137.9–139.7)	138.2 (137.3–139.1)	138.3 (137.4–139.1)	138.8 (137.9–139.7)	139.1 (138.2–139.9)	138.9 (138.0–139.8)		
Group 3	138.2 (137.3–139.1)	138.7 (137.9–139.6)	140.1 (139.2–140.9)	140.7 (139.9–141.6)	141.4 (140.5–142.2)	140.8 (139.9–141.7)	140.1 (139.2–140.9)	139.1 (138.2–139.9)	139.0 (138.1–139.9)		
Group 4	138.9 (138.0–139.8)	137.9 (137.0–138.8)	137.5 (136.7–138.4)	137.8 (136.9–138.7)	138.2 (137.3–139.1)	138.4 (137.5–139.2)	138.4 (137.5–139.2)	138.0 (137.1–138.9)	138.1 (137.2–138.9)		
*p*^‡^	0.71	<0.001	<0.001	<0.001	<0.001	<0.001	<0.001	<0.001	<0.001		

Data are presented as mean (95% CI) at baseline, and least-squares mean (95% CI) at 1 h, 2 h, 3 h, and 4 h, respectively

Analysis was conducted using a mixed model for repeated measures, adjusting for baseline values

*p Value for time effect—trend over time in all arms

^†^*p* Value for treatment × time interaction—evaluates if changes in one arm are different from the changes in the other arms

^‡^*p* Value for comparison between arms at each moment

### The effect of interventions on PRA and aldosterone

As observed in Table 5, there were no significant differences between the 4 groups regarding PRA and aldosterone at baseline. Considering all 4 groups, there was a significant increase in the mean concentration (Table 5, p* < 0.001) and a significant difference in the slope between the 4 groups (Table 5, *p*^†^ < 0.001).

**Table 5:** The effect of interventions on aldosterone and plasma renin activity (PRA)

	Length of follow-up		p*	*p*^†^
	Baseline	120 min		
Aldosterone, ng/dL				
Group 1	56.1 (51.3–60.9)	64.7 (59.9–69.6)	<0.001	<0.001
Group 2	59.2 (54.3–64.0)	56.6 (51.8–61.5)		
Group 3	52.0 (47.2–56.9)	56.4 (51.6–61.3)		
Group 4	58.8 (53.9–63.6)	57.4 (52.6–62.3)		
* p*^‡^	0.15	<0.001		
PRA, ng/mLh				
Group 1	1.7 (1.4–2.0)	2.3 (2.0–2.6)	<0.001	<0.001
Group 2	1.4 (1.1–1.7)	1.4 (1.1–1.7)		
Group 3	1.4 (1.0–1.7)	1.7 (1.3–1.9)		
Group 4	1.2 (0.9–1.6)	1.3 (0.9–1.6)		
* p*^‡^	0.25	<0.001		

Data are presented as mean (95% CI) at baseline, and least-squares mean (95% CI) at 2 h

Analysis was conducted using a mixed model for repeated measures, adjusting for baseline values

*p Value for time effect—trend over time in all arms

^†^*p* Value for treatment × time interaction—evaluates if changes in one arm are different from the changes in the other arms

^‡^*p* Value for comparison between arms at each moment

**Fig. 5:**
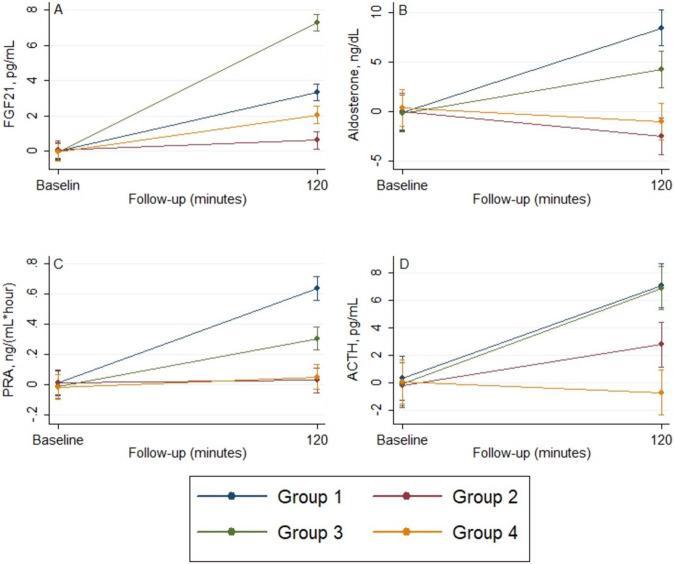
The mean values of serum parameters across the follow-up. **A** Fibroblast growth factor 21 (FGF21). **B** Aldosterone. **C** Plasma renin activity (PRA). **D** Adrenocorticotropic hormone (ACTH)

The mean values were increased in Groups 1 and 3 for aldosterone and PRA values (Fig. 5B, C). At 120 min of assessment, Group 1 had significantly increased levels of aldosterone and PRA than those from all the other groups. Individuals from Group 3 had higher levels of aldosterone and PRA than those in Groups 2 and 4.

### The effect of interventions on ACTH and cortisol

At baseline, there were no significant differences between the 4 groups regarding ACTH and cortisol (Table 6). Considering the 4 groups there was a significant increase in the mean concentration (Table 6, p* < 0.001) and a significant difference in the slope between the 4 groups (Table 6, *p*^†^ < 0.001).

**Table 6:** The effect of interventions on serum adrenocorticotropic hormone (ACTH) and cortisol

	Length of follow-up		p*	*p*^†^
	Baseline	120 min		
Cortisol, mcg/dL				
Group 1	18.1 (15.9–20.2)	27.3 (25.2–29.5)	<0.001	<0.001
Group 2	17.5 (15.4–19.7)	19.4 (17.3-21.6)		
Group 3	18.6 (16.4–20.7)	25.4 (23.3–27.6)		
Group 4	19.3 (17.2–21.4)	22.1 (19.9–24.3)		
*p*^‡^	0.71	<0.001		
ACTH, pg/mL				
Group 1	21.2 (18.4–23.9)	27.9 (25.1–30.7)	<0.001	<0.001
Group 2	17.1 (14.3–19.9)	20.1 (17.3–22.9)		
Group 3	20.1 (17.3–22.8)	27.1 (24.3–29.8)		
Group 4	21.6 (18.8–24.3)	20.8 (18.0–23.6)		
* p*^‡^	0.11	<0.001		

Data are presented as mean (95% CI) at baseline, and least-squares mean (95% CI) at 2 h

Analysis was conducted using a mixed model for repeated measures, adjusting for baseline

*p Value for time effect—trend over time in all arms

^†^*P* Value for treatment × time interaction—evaluates if changes in one arm are different from the changes in the other arms

^‡^*p* Value for comparison between arms at each moment

The mean values were increased in all groups for cortisol, only in Groups 1, 2, and 3 for ACTH (Fig. 5D). At 120 min of assessment, individuals in Groups 1 and 3 had significantly higher mean values than individuals in Groups 2 and 4 for ACTH levels. For cortisol values, individuals in Group 1 had significantly increased levels than those from all the other groups, while individuals in Group 3 had higher levels than those in Groups 2 and 4 (Fig. 3D).

### The effect of interventions on serum glucose concentrations

At baseline, individuals from Group 4 had higher glucose levels than individuals from Groups 1 and 2, while individuals from Group 3 only than those from Group 1 (Table 7). Considering all the groups, during the evaluation, there was a significant increase in the mean plasma sodium concentration (Table 7, p* < 0.001) and a significant difference in the slope between the four groups (Table 7, *p*^†^ < 0.001). As compared with the baseline values, in individuals from Group 3, there was an increase in mean glucose values starting from the first assessment point by steady concentrations afterward, while in individuals from Group 4, this increase was significant only from 30 to 75 min of follow-up (Fig. 4A). Individuals from Group 3 had higher mean glucose values at all time points, and only in the first 105 min and 90 min than individuals from Group 1, 2 and 4, respectively (Fig. 4A).

**Table 7:** The effect of interventions on serum glucose concentrations

	Length of follow-up									p*	*p*^†^
	Baseline	15 min	30 min	45 min	60 min	75 min	90 min	105 min	120 min		
Glucose, mg/dL											
Group 1	72.3 (65.2–79.4)	88.6 (81.5–95.7)	88.6 (81.5–95.7)	70.9 (63.9–78.1)	74.6 (67.5–81.7)	77.2 (70.1–84.3)	78.0 (70.9–85.1)	77.2 (70.1–84.3)	75.5 (68.4–82.6)	<0.001	<0.001
Group 2	75.2 (68.1–82.3)	84.8 (77.7–91.9)	79.4 (72.3–86.5)	75.8 (68.7–82.9)	75.6 (68.5–82.7)	77.0 (69.9–84.1)	79.5 (72.4–86.6)	78.9 (71.8–86.0)	82.3 (75.2–89.4)		
Group 3	87.5 (80.4–94.6)	116.9 (109.8–124.0)	128.9 (121.8–136.0)	126.1 (118.9–133.2)	121.3 (114.2–128.4)	114.5 (107.4–121.6)	112.2 (105.1–119.3)	107.1 (99.9–114.2)	100.5 (93.4–107.6)		
Group 4	88.8 (81.7–95.9)	99.1 (91.9–106.2)	103.7 (96.6–110.8)	104.0 (96.9–111.1)	103.1 (95.9–110.2)	101.3 (94.2–108.4)	99.4 (92.3–106.5)	97.5 (90.4–104.6)	95.5 (88.4–102.6)		
*p*^‡^	0.001	<0.001	<0.001	<0.001	<0.001	<0.001	<0.001	<0.001	0.02		

Data are presented as mean (95% CI) at baseline, and least-squares mean (95% CI) at 15 min, 30 min, 45 min, 60 min, 75 min, 90 min, 105 min, and 120 min, respectively

Analysis was conducted using a mixed model for repeated measures, adjusting for baseline values

*p Value for time effect—trend over time in all arms

^†^*p* Value for treatment × time interaction—evaluates if changes in one arm are different from the changes in the other arms

^‡^*p* Value for comparison between arms at each moment

### The effect of interventions on uric acid and FGF21

The mean values were increased in Groups 1, 3, and 4 for FGF21 and Groups 1 and 3 for uric acid (Table 8, *p* < 0.001). At 120 min of assessment, individuals in Groups 1 and 3 had significantly higher mean values than those in Groups 2 and 4 for uric acid levels (Fig. 3C). For FGF21 values, individuals from Group 3 had significantly higher levels than those from all the other groups and individuals from Group 1 had only than those from Groups 2 and 4 (Fig. 5A).

**Table 8:** The effect of interventions on Uric acid and fibroblast growth factor 21 (FGF21)

	Length of follow-up		p*	*p*^†^
	Baseline	120 min		
Uric acid, mg/dL				
Group 1	5.2 (4.5–5.8)	5.4 (4.8–6.0)	<0.001	<0.001
Group 2	5.3 (4.6–5.9)	5.2 (4.6–5.9)		
Group 3	5.2 (4.6–5.8)	5.5 (4.9–6.2)		
Group 4	5.2 (4.6–5.8)	5.2 (4.6–5.8)		
* p*^‡^	0.99	<0.001		
FGF21, pg/mL				
Group 1	10.1 (9.3–10.8)	13.4 (12.6–14.2)	<0.001	<0.001
Group 2	10.7 (9.9–11.5)	11.2 (10.5–12.0)		
Group 3	10.2 (9.4–10.9)	17.5 (16.7–18.2)		
Group 4	9.9 (9.2–10.7)	12.0 (11.3–12.8)		
* p*^‡^	0.52	<0.001		

Data are presented as mean (95% CI) at baseline, and least-squares mean (95% CI) at 2 h

Analysis was conducted using a mixed model for repeated measures, adjusting for baseline values

*p Value for time effect—trend over time in all arms

^†^*p* Value for treatment × time interaction—evaluates if changes in one arm are different from the changes in the other arms

^‡^*p* value for comparison between arms at each moment.

## Discussion

In this study, we investigated the impact of fructose and salt intake on the initial physiological alterations, metabolic indicators, and regulators of sodium metabolism. Additionally, we explored whether water plays a protective role in counteracting the metabolic effects of fructose consumption. Consistent with our hypothesis, the findings revealed that the consumption of fructose and salt leads to elevated BP, osmolarity, and metabolic markers including uric acid, cortisol, FGF21, aldosterone, PRA, and ACTH. While salt had a greater impact on RAS and BP, fructose consumption caused a greater rise in serum glucose and FGF21. We also demonstrated that supplemental water intake ameliorated the metabolic effects of fructose, further underscoring its potential protective role.

Our study revealed that serum and urine osmolarity increased due to fructose consumption without water, similar to salt consumption, which was also demonstrated in previous studies [15–18]. Fructose induces the shift of water into the cell for rapid synthesis of glucose and glycogen, resulting in hyperosmolarity [5, 8, 19]. In addition, apple juice osmolality can reach up to 696 mOsm/kg [20]. Fructose is slowly absorbed along the small intestine through passive carrier-mediated facilitated diffusion (GLUT 5), preventing the absorption of trapped water in this compartment and inducing a transient shift of fluid into the small intestine [21]. The increased sodium level in response to fructose consumption that was observed in our study may also be due to salt absorption from the intestines and the proximal tubules of the kidneys [22]. These various mechanisms by which fructose can increase osmolality may also explain why this sugar induces vasopressin more significantly than glucose [23].

Osmoreceptors detect changes in serum osmolarity rapidly due to their location outside the blood-brain barrier, thus, we observed a very robust response to small osmolarity alterations in this study [24–27]. Increased serum osmolarity causes an acute stress response via several mechanisms, including the direct effect of vasopressin, secondary activation of RAS, activation of the sympathetic nervous system, and increased synthesis of ACTH which explains the findings of this study [28]. Increased serum osmolarity also causes activation of the aldose-reductase pathway generating sorbitol to protect the renal tubular cells from the high osmolarity of the extracellular environment [24, 29]. However, the sorbitol can further be degraded to fructose and metabolized by fructokinase, causing ATP depletion and generation of uric acid, inflammation, oxidative stress, and vasoactive substances [29–31].

In this study, salt consumption had a more profound impact on RAS and BP than fructose, whereas ACTH and uric acid induction were similar. Although the activation of RAS results seems counterintuitive due to the negative feedback mechanism of plasma sodium, it should be noted that we measured plasma PRA and aldosterone only after the first 2 h. Aldosterone secretion depends on various mechanisms, including vasopressin and ACTH, which could be why we detected an increase in PRA and aldosterone in the first 2 h [32]. Indeed, individuals are asked to consume a high sodium load in diagnosing primary hyperaldosteronism with oral sodium load, and aldosterone is measured on the final day from a 24-h urine specimen whereas our study represents only an early response to oral salt and fructose consumption [33]. Fructose consumption increased the PRA and aldosterone levels only when administered without water in a similar pattern to salt, although to a lesser extent. This finding reveals that fructose induction of aldosterone is mediated through vasopressin, and when vasopressin is suppressed by water, RAS activation is not observed [34].

The similar pattern of fructose and salt consumption of uric acid demonstrates the endogenous production of fructose synthesis and metabolism in response to serum osmolarity. The studies that investigated the relationship between sodium intake and serum uric acid concentration for one week to one month found increased sodium intake to be inversely correlated with serum uric acid level, possibly due to diuresis in proximal tubules [35, 36]. However, our results demonstrate the acute process; the rapid effect of serum osmolarity possibly predominates the early metabolic changes. Although uric acid is an antioxidant in plasma, once inside the vascular smooth muscle cells, endothelial cells, and adipocytes, it inhibits nitric oxide production, induces platelet activation and a pro-inflammatory state, and causes endothelial dysfunction [37, 38]. Uric acid is considered to be a risk factor for metabolic syndrome, diabetes, kidney disease, and cardiovascular disorders [37]. By inducing the RAS, diminishing nitric oxide in the endothelium, and decreasing renal perfusion, uric acid also contributes to hypertension [39, 40]. The uric acid did not increase in the group that drank water while consuming fructose, suggesting that water may serve as a protective factor against the adverse metabolic effects induced by uric acid resulting from fructose intake.

Our results highlight that in addition to increased osmolarity, fructose consumption also induces metabolic hyperglycemia, demonstrated by increased plasma glucose, which was diminished with hydration. FGF21, a stress-inducible circulating protein synthesized by the liver, followed the same pattern as hyperglycemia. FGF21 is synthesized in response to carbohydrate absorption and regulates insulin sensitivity and lipid and energy metabolism [41]. FGF21 is postulated to play an adaptive role in response to physiologic and metabolic stressors and is found elevated in poor metabolic health, including obesity [42], type 2 diabetes mellitus [43], and insulin resistance [44]. As seen from the other parameters in our study, water decreases fructose’s effect on metabolism and possibly prevents a stress response from overnutrition, which results in a decreased FGF21 response. Given that the clinical outcomes of sugar-sweetened beverages such as type-2 diabetes, cardiovascular diseases, and non-alcoholic fatty liver diseases, are not solely a result of increased caloric intake but also due to various metabolic processes, hydration can also be essential in preventing these long-term consequences [45]. FGF21 levels in humans have been shown to increase following sugar consumption, with sucrose being a potent inducer. These elevations suggest a regulatory role of FGF21 on sugar intake, supported by genetic studies linking variants in the FGF21 gene to carbohydrate consumption preferences [46]. Notably, FGF21 also moderates alcohol intake, indicating its broad regulatory effects on sugar and related substances [47].

The acute stimulation of FGF21 following fructose ingestion is dose-dependent, peaking at about 2 h post-consumption, and is highly reproducible within individuals, hinting at its potential as a metabolic regulator in response to diet [48]. This response is robust even at lower doses of fructose, and the ratio of intact to total FGF21 remains stable, underscoring the hormone’s consistent regulatory behavior in the presence of fructose. These findings are significant given the prevalence of fructose in the modern diet and its association with metabolic diseases. The clear dose-dependent response of FGF21 to fructose and its reproducibility make it a promising marker for dietary sugar intake and metabolic health assessment. We also demonstrated that, although less than the fructose consumption, salt consumption increased FGF21 as well. In an animal model of hypertension, angiotensin-II induces FGF21, which has a compensatory response to hypertension by angiotensin-converting enzyme II, which inactivates angiotensin-II [49]. In the context of salt-sensitive hypertension, which is frequently associated with severe kidney damage and progression to end-stage kidney disease, FGF21 has been recognized as a suppressor of nephropathy in diabetic mice models. It was shown that a significant increase in both circulating levels and renal expression of FGF21 in hypertensive mice was induced by deoxycorticosterone acetate-salt treatment [50]. Our study demonstrated an increase in the RAS pathway in the first 2 h, which could account for the increase in FGF21.

One of the key findings in this study is the mitigating effect of water on the early metabolic effects of fructose. In 2020, an animal model demonstrated the vasopressin modulation in fructose-fed mice and the metabolic syndrome that arose from this pathway after 30 weeks; when enough hydration to suppress vasopressin was maintained, the metabolic syndrome did not arise in the mice [5]. Furthermore, lowering vasopressin in already obese mice ameliorated the metabolic effects of glucose, as well demonstrated by improvement in fatty liver, hyperinsulinemia, hyperleptinemia, and adipose inflammation [5]. In a previous study of our group, we demonstrated that fructose consumption via apple juice increases the level of copeptin, a marker of increased vasopressin [16]. Unsurprisingly, cohort and cross-sectional studies demonstrated that increased water intake is associated with lower vasopressin [51]. Considering the metabolic effects of vasopressin, including increased BP, induction of RAS, and increased expression of ACTH [34], we postulate that the mitigation of early metabolic impacts by hydration is due to decreased vasopressin secretion. The prevention of increased BP by concurrent water administration is also in accordance with our previous study conducted in 2018, by which we found that the changes in BP can be prevented by decreasing serum osmolarity and vasopressin levels when the salty soup was administered with additional water [12]. Therefore, other than limiting salt intake and sugary beverages, increased water consumption can be advised to individuals and the general population.

If this study had been conducted on hypertensive individuals instead of healthy volunteers, the findings could potentially be different. Given that hypertension is associated with changes in renal function and sodium handling, the subjects might have exhibited a more pronounced physiological response to sodium and fructose intake. This could be reflected in altered serum osmolality and metabolic responses, possibly with greater increases in BP and more significant alterations in metabolic parameters like FGF21 levels. Furthermore, the role of hydration in mitigating these effects might be more critical in hypertensive patients, who often have impaired sodium excretion.

The limitations of this study include the small sample size in each group, which decreases the power to detect differences. Also, the participants in Group 4 were exposed to interventions comprising 500 mL of 100% apple juice and 500 mL of water administered within a 5-min timeframe. This led to a cumulative fluid intake of 1000 mL, exhibiting a twofold difference compared to the other study groups. It is imperative to explicitly recognize and address this discrepancy as a limitation inherent in the study design. We also used 100% apple juice to evaluate the fructose effect on metabolic changes to mimic the real-life consumption of sugary beverages. To minimize potential confounding factors, we opted for apple juice over citrus juice, which has minimal known antioxidant properties. However, other components in apple juice might have had direct or indirect effects on the biomarkers under investigation. The metabolic effects we investigated are only short-term effects. Future longitudinal studies are needed for the long-term consequences of fructose and water consumption and whether hydration significantly mitigates fructose-induced risk of metabolic syndrome.

### Perspective of Asia

Our findings offer valuable insights for health professionals in Asia, especially regarding the early metabolic effects of sodium and fructose intake and the formulation of intervention strategies. For instance, emphasizing hydration could be integrated more prominently into regional dietary habits and lifestyle, aiding individuals in developing healthy drinking habits from an early age and mitigating potential health risks.

The varying geographical and climatic conditions in Asia play a significant role in the maintenance of water and electrolyte balance. Individuals living in hot and humid climates are particularly advised to increase their daily fluid intake to reduce the risk of dehydration and maintain metabolic health. Our study highlights the potential protective effects of hydration for individuals in these climatic conditions. Furthermore, the results of our study could serve as a crucial resource in the process of developing health policies and clinical practices in Asia, enhancing awareness of the health effects of salt and sugar intake, and informing public health strategies. Taking into consideration regional characteristics and demographic factors, such research can improve the understanding of health issues in Asia and contribute to the development of effective intervention methods.

## Conclusion

The consumption of 100% apple juice and salt triggers a robust stress response characterized by increased BP, serum and urine osmolarity, glucose, sodium, uric acid, ACTH, cortisol, FGF21, aldosterone, and PRA. RAS activation. The increase in BP was more prominent with salt intake, whereas plasma glucose and FGF21 levels were elevated in response to fructose intake. The effects on ACTH, cortisol, and uric acid were similar. We postulate that even a slight rise in serum osmolarity may trigger a vasopressin response that modulates this similar metabolic pattern. Hydration almost entirely prevented the metabolic response to fructose, which can be explained by preventing the increase in osmolarity and vasopressin response. It is critical in further research and clinical practice to emphasize understanding the role of serum osmolarity and hydration. Current guidelines should not only mention the dietary amount of sodium and fructose, how it is consumed, and how much hydration individuals can maintain in their daily lives.

In conclusion, 100% apple juice and salt consumption caused a robust stress response characterized by increased BP, serum and urine osmolarity, glucose, sodium, uric acid, ACTH, cortisol, FGF21, aldosterone, and PRA. RAS activation. The BP increase was more prominent with salt intake, whereas plasma glucose and FGF21 increased in response to fructose intake. The effect on ACTH, cortisol, and uric acid was similar. We postulate that even a slight rise in serum osmolarity may result in a vasopressin response that modulates this similar metabolic pattern. Hydration almost completely prevented the metabolic response to fructose, which can be explained by the prevention of the increase in osmolarity and vasopressin response. In further research and clinical practice, it is critical to emphasize understanding the role of serum osmolarity and hydration. Current guidelines should not only mention the dietary amount of sodium and fructose, how it is consumed, and how much hydration people can maintain in their daily lives.
